# Blood Mononuclear Cell Mitochondrial Respiratory Chain Complex IV Activity is Decreased in Multiple Sclerosis Patients: Effects of β-Interferon Treatment

**DOI:** 10.3390/jcm7020036

**Published:** 2018-02-20

**Authors:** Iain Hargreaves, Nimesh Mody, John Land, Simon Heales

**Affiliations:** 1 Neurometabolic Unit, National Hospital, Queen Square, London WC1N 3BG, UK; n.mody@abdn.ac.uk (N.M.); j.land@ucl.ac.uk (J.L.); 2 Department of Pharmacy and Biomolecular Science, Liverpool John Moores University, Byrom Street, Liverpool L3 5UA, UK; 3 Institute of Medical Sciences, University of Aberdeen, Scotland AB24 3FX, UK; 4 UCL Great Ormond Street Institute of Child Health, University College London, London WC1E 6BT, UK

**Keywords:** mitochondrial respiratory chain, complex IV, blood mononuclear cells, multiple sclerosis, β-Interferon

## Abstract

Objectives: Evidence of mitochondrial respiratory chain (MRC) dysfunction and oxidative stress has been implicated in the pathophysiology of multiple sclerosis (MS). However, at present, there is no reliable low invasive surrogate available to evaluate mitochondrial function in these patients. In view of the particular sensitivity of MRC complex IV to oxidative stress, the aim of this study was to assess blood mononuclear cell (BMNC) MRC complex IV activity in MS patients and compare these results to age matched controls and MS patients on β-interferon treatment. Methods: Spectrophotometric enzyme assay was employed to measure MRC complex IV activity in blood mononuclear cell obtained multiple sclerosis patients and aged matched controls. Results: MRC Complex IV activity was found to be significantly decreased (*p* < 0.05) in MS patients (2.1 ± 0.8 k/nmol × 10^−3^; mean ± SD] when compared to the controls (7.2 ± 2.3 k/nmol × 10^−3^). Complex IV activity in MS patients on β-interferon (4.9 ± 1.5 k/nmol × 10^−3^) was not found to be significantly different from that of the controls. Conclusions: This study has indicated evidence of peripheral MRC complex IV deficiency in MS patients and has highlighted the potential utility of BMNCs as a potential means to evaluate mitochondrial function in this disorder. Furthermore, the reported improvement of complex IV activity may provide novel insights into the mode(s) of action of β-interferon.

## 1. Introduction

Multiple sclerosis (MS) is an inflammatory demyelinating disease of the central nervous system (CNS), in which cytokines and other inflammatory mediators are raised [1]. To date, the exact cause of MS has still to be fully elucidated, but it is believed to result from an abnormal response of the immune system to one or more myelin antigens in the CNS, such as components of the myelin [2]. The disease is characterized by an accumulation of macrophages and lymphocytes in the CNS leading to demyelination and destruction of neuronal axon [3]. These areas of demyelination are known as plaques and contain areas of gliosis and inflammation in most cases [4].

MS has a heterogeneous clinical presentation with symptoms including impaired vision, fatigue, spasms and paralysis of a number of muscle systems [5]. There are five basic types of MS of which relapsing remitting (RR) is the most common [6]. In RR-MS patients the disease develops in a discursive manner with symptomatic and asymptomatic phases. Over time, RR-MS patients may develop chronic lesions that result in irreversible axonal damage and loss, resulting in the conversion of RRMS to secondary progressive MS (SPMS).

Although MS is traditionally considered to be an autoimmune disease, neurodegeneration has also been implicated in disease progression [7]. One of the major factors that are responsible for neurodegeneration in MS is thought to be mitochondrial respiratory chain (MRC) dysfunction with evidence of impaired MRC complex I (NADH: ubiquinone reductase; EC: 1.3.5.1), III (Ubiquinol: cytochrome reductase; EC: 1.10.2.2.), and IV (Cytochrome c oxidase; EC: 1.9.3.1) activities being reported in post mortem cerebral tissue from MS patients, as well as in experimental autoimmune encephalomyelitis [8,9,10]. Although the cause of the MRC dysfunction in MS has still to be fully elucidated, oxidative and nitrosative stress are thought to be contributory factors [10]. Reactive oxygen species (ROS), such as superoxide (O_2_^-^) and reactive nitrogen species (RNS: nitric oxide (NO); peroxynitrite; ONOO^-^) are generated during neuro-inflammation in MS and have been implicated, by our research and that of others, as mediators of demyelination and axonal injury [11,12,13,14]. Although the inflammatory environment in demyelinating plaques is conducive to the generation of ROS, activated lymphocytes and macrophages also release a host of pro-inflammatory cytokines, such as interferon gamma (IFN-g), which results in an upregulation inducible nitric oxide synthase (iNOS) activity within the CNS and a concomitant increase RNS generation [15]. ROS and RNS are able to induce MRC dysfunction by causing oxidative damage to mitochondrial DNA, mitochondrial membrane phospholipids, and/or the protein subunits of the enzymes [16]. The continued inflammatory process in the CNS of MS patients coupled to the impaired immune regulation results in high circulatory levels of RNS [17], which may have the potential to impair MRC function in peripheral tissue. Although few studies have assessed this phenomenon, a study by Kumleh et al. [18] reported evidence of impaired skeletal muscle MRC complex I activity in a small cohort of MS patients. Although it is difficult in living MS patients to accurately determine evidence of cerebral MRC dysfunction, the presentation of mitochondrial dysfunction in systemic tissue may provide an appropriate surrogate for this evaluation. The liberation of a skeletal muscle biopsy, which is considered the “gold standard” for MRC enzyme determination [19] would be relatively invasive and may not always be possible. However, the use of blood mononuclear cells (BMNCs) may provide an alternative relatively low invasive means to assess the evidence of MRC dysfunction in MS patients. Furthermore, in view of the relatively small amount of biological material afforded from a BMNC preparation it would not be possible to assess the activity of all the MRC enzyme complexes. However, in view of the particular susceptibility of MRC complex IV activity to RNS induced inactivation [19], assessment of the activity of this enzyme complex may therefore be judicious in MS patients. Furthermore, this tissue may also be informative with regards to the efficacy and mode of action of therapeutic agents, such as β-interferon, which may slow disease progression. 

The purpose of this study was therefore to determine BMNC complex IV activity in MS patients and compare these results to age matched controls and MS patients receiving therapy in the form of β-interferon.

## 2. Experimental Section

### 2.1. Reagents

All of the reagents were analytical grade and obtained Sigma Aldrich Chemical Company (Poole, Dorset, UK). PD_10_ column used in the preparation of reduced cytochrome c for complex IV spectrophotometric enzyme assay were obtained from Amersham Pharmacia (St. Albans, Herts, UK).

### 2.2. Patients

Patients were diagnosed and consented by a consultant neurologist at the National Hospital, Queen Square, London, UK, according to the guidelines of Poser et al. [20]. They were divided into two groups:

(1) Patients not receiving β-interferon treatment. This group consisted of seven patients (male:female = 4:3). Six patients were aged between 30–39 years and one female patient was aged 65 years.

(2) Patients receiving β-interferon treatment. This group consisted of four patients (male:female = 3:1). Patients were aged between 26-36 years. Patients were selected and received β-interferon in accordance with the National Institute for Health and Care Excellence (UK) guidelines.

For this study, a control group of 24 healthy volunteers (aged 32–55 years, male:female = 9:15) were used.

### 2.3. Blood Mononuclear Cell (BMNC) Preparation

BMNCs were isolated from between 3–10 mL of lithium heparin blood by use of the ACCUSPIN system–Histopaque-1077 (Sigma-Aldrich, Poole, Dorset, UK). BMNCs were suspended in phosphate buffered saline, pH 7.2, and stored at –70 °C until analysis.

### 2.4. Spectrophotometric Enzyme Assays

Enzymatic determinations were undertaken at 30 °C using a Uvikon XL spectrophotometer (Northstar, Leeds, UK).

MRC complex IV activity was measured by the potassium cyanide sensitive oxidative of reduced cytochrome c at 550 nm, according to the method of Wharton and Tzagoloff [21].

To account for the mitochondrial enrichment of the preparations used, activity of the mitochondrial marker enzyme, citrate synthase (CS) (EC 2.3.3.1) was evaluated. This was determined according to the method of Shepherd and Garland [22] by the formation of 5-thio-2-nitrobenzoic following the incubation BMNCs with acetyl-CoA, oxaloacetate, and 5,5-Dithiobis-(2-nitrobenzoic acid). 5-thio-2-nitrobenzoic absorbs at 412 nm. 

Complex IV activities were expressed as a ratio to CS activity (k/nmol) to take into account the mitochondrial enrichment of the BMNCs [23].

CS has units of activity of nmol/min/mL. Complex IV has units of activity of k/min/mL since the activity of this enzyme is expressed as a 1st order rate constant. Therefore, when complex IV activity is expressed as a ratio to CS activity the units are: k/min/mL divided by nmol/min/mL = k/nmol.

### 2.5. Protein Determination

Protein was determined according to the method of Lowry and colleagues [24] using bovine serum albumin as a standard.

### 2.6. Statistical Analysis

Statistical analysis was performed using one-way analysis of variance (ANOVA) followed by the least squared difference (LSD) multiple range test, the students *t*-test, and Spearman test was used to establish potential correlations between MRC complex IV activity, CS activity, and age. A *p* value < 0.05 was considered to be statistically significant.

## 3. Results

Recombinant β-interferon (4 and 16 million units) was not found to have an effect on MRC complex IV or CS activities in vitro. No correlation was found between age and BMNC MRC complex IV (*r* = 0.688; *n* = 21; *p* = 0.7703) or CS (*r* = –0.276; *n* = 21; *p* = 0.742) activities, respectively, in the control population. Gender was also not found to influence the activities of these enzymes in BMNCs, with no significant difference being found between male and female complex IV (*p* = 0.675) or CS (*p* = 0.691) activities.

**Figure 1:**
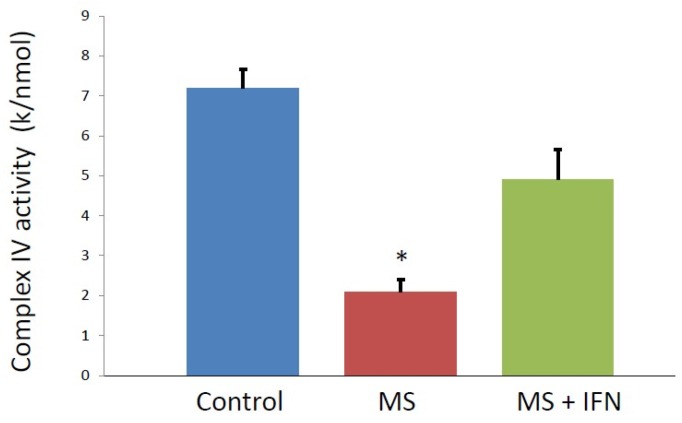
Blood mononuclear cell Complex IV activity, expressed as a ratio to citrate synthase, in control individuals, MS patients and MS patients receiving β-interferon (IFN). * Statistically different from both control and MS patients receiving β-interferon.

BMNC MRC complex IV activity (expressed as a ratio to CS activity) was found to be significantly decreased (*p* < 0.05) in MS patients not on β-interferon (2.1 ± 0.8 k/nmol × 10^−3^; mean ± SD) when compared to the controls (7.2 ± 2.3 k/nmol × 10^−3^) (Figure 1). Complex IV activity in MS patients on β-interferon (4.9 ± 1.5 k/nmol × 10^−3^) was not found to be significantly different from that of the controls (Figure 1). No significant difference in BMNC CS activity was found between the control (45.24 ± 18.77 nmol/min/mg) and MS patients (33.65 ± 10.02 nmol/min/mg).

## 4. Discussion

The results of this study have indicated evidence of a deficiency in MRC complex IV activity in BMNCs of MS patients. The impairment of BMNC MRC complex IV activity may result in an altered immune response, which may contribute to disease pathophysiology. At present, the factors responsible for this MRC dysfunction in the MS patients are as yet uncertain. However, the absence of a significance decrease in BMNC MRC complex IV activity in MS patients receiving β-interferon suggests that the loss of enzyme activity may be the result of a disease process that is reversed by β-interferon. One of the mechanisms of action by which β-interferon elicits its beneficial effect in MS patients appears to be by its ability to inhibit astrocytic NO production [21], and thereby decreasing the availability of circulatory RNS that have the potential to induce MRC impairment, particularly at the level of complex IV. Whether such a mechanism occurs in the periphery requires further investigation. However, it is of note that serum levels of nitrite and nitrate (indices of RNS production) are reported to be elevated in MS patients [17]. Alternatively, the decrease in MRC complex IV activity detected in the MS patients may be the result of mitochondrial DNA deletions as reported in the neurons and choroid plexus of progressive MS patients [22]. Although, evidence of mitochondrial DNA mutations, and effects of β-interferon, in peripheral BMNCs has yet to be determined in MS patients [23]. Nonetheless, a study by Amorini el al. has reported a threefold elevation in serum lactate levels in MS patients [24]. Although this study supports evidence of mitochondrial dysfunction in MS, previous studies assessing both serum [25] and CSF (cerebral spinal fluid) [26] lactate levels in this disorder have failed to show any evidence of an increase in the level of this metabolite. Importantly, lactate levels may not necessarily be raised as a consequence of MRC dysfunction as evidenced in patients with primary mitochondrial disorders [27]. Furthermore, elevated serum lactate levels may not be a specific biomarker of MRC dysfunction, since this phenomenon has been reported to result from number of other clinical sequelae [27]. Therefore, the determination of MRC complex IV activity in BMNCs may serve as a more specific means of evaluating evidence of MRC dysfunction in MSA patients. In addition, in view of the association between oxidative and nitrosative stress and MRC dysfunction [11,12,13,14], BMNCs may also serve as a means of assessing the cellular antioxidant status of MS patients. In view of its association with MRC dysfunction, the status of the cellular antioxidant, reduced glutathione may be judicious for this assessment [28]. The possibility arises that MRC complex IV dysfunction may also be a contributory factor to the pathophysiology of other diseases that are associated with nitrosative stress, such as diabetes, cancer, and stroke [29,30,31], and therefore, BMNCs may have utility is assessing evidence of mitochondrial impairment in these disorders.

In this study we have highlighted the feasibility of using BMNCs to assess evidence of MRC complex IV deficiency in MS patients. However, due to the limited amount of biological material available from BMNCs, it has not been possible to determine the activities of the other MRC enzymes (complexes I, II, and III) in the present study, and therefore, we cannot exclude the possibility that the MRC dysfunction in the MS patients is not solely restricted to complex IV. In spite of its limitations, this is the first study as far as the authors are aware to use BMNCs are a relatively low invasive surrogate to assess evidence of mitochondrial dysfunction in MS. This surrogate may also be of value in monitoring the therapeutic potential of pharmacotherapies on mitochondrial function in MS patients in view of the paucity of reliable biomarkers that are currently available.
